# *Sarcocystis* spp. Macrocysts Infection in Wildfowl Species in Eastern Baltic Region: Trends in Prevalence in 2011–2022

**DOI:** 10.3390/ani13182875

**Published:** 2023-09-10

**Authors:** Petras Prakas, Jolanta Stankevičiūtė, Saulius Švažas, Evelina Juozaitytė-Ngugu, Dalius Butkauskas, Rasa Vaitkevičiūtė-Balčė

**Affiliations:** 1 Nature Research Centre, Akademijos 2, 08412 Vilnius, Lithuania; saulius.svazas@gamtc.lt (S.Š.); evelina.ngugu@gamtc.lt (E.J.-N.); dalius.butkauskas@gamtc.lt (D.B.); 2 Agriculture Academy, Vytautas Magnus University, Studentų Str 11, Kaunas District, 53361 Akademija, Lithuania; jolanta.stankeviciute1@vdu.lt (J.S.); rasa.vaitkeviciute1@vdu.lt (R.V.-B.)

**Keywords:** ducks, hunting, macrocysts, *Sarcocystis rileyi*, infection rates, long-term monitoring

## Abstract

**Simple Summary:**

Macrocysts of the protozoan *Sarcocystis rileyi*, resembling rice grains, are found in the muscles of numerous duck species. Meat from ducks contaminated with *S*. *rileyi* is not suitable for consumption, and severe *S*. *rileyi* infections are harmful to infected birds. This *Sarcocystis* species was reported only in North America for a long time. However, recently, the number of reports of *S. rileyi* infection in European wild ducks has increased. The present research is the first long-term surveillance study on macrocysts in wildfowl species. Overall, 3268 individuals of wildfowl species collected in Lithuania, Latvia, Russia, and Belarus were tested for macrocysts of *Sarcocystis*. The examined parasite was detected in 237 Mallards and in two Eurasian Teals. Macrocysts isolated from 37 Mallards were examined by DNA methods and identified as *S*. *rileyi*. The infection rates of macrocysts in Mallards in the examined region ranged from 3.1% to 8.7%. The fluctuations in infection rates were observed in different years, months, and studied countries. Based on the results of the current research and previous studies, the prevalence of macrocysts depends on the duck species, age of the bird, year, geographical region, and hunting season but is not related to the sex of the bird.

**Abstract:**

Wildfowl meat infected with *S*. *rileyi* macrocysts is not suitable for human consumption. Ducks are among the main game birds in Europe, and *S*. *rileyi* infections cause significant economic losses. In 2011–2022, a total of 2649 anseriforms collected in Lithuania and 619 Mallards (*Anas platyrhynchos*) hunted in the Kaliningrad region of Russia, Belarus, and Latvia were tested for macrocysts. In Lithuania, macrocysts were detected in 206 of 2362 Mallards (8.7%) and in two of 88 (2.3%) Eurasian Teals (*Anas crecca*). The prevalence of macrocysts in the other three countries, Belarus (5.9%), Russia (5.0%), and Latvia (3.1%), was similar. For species identification, macrocysts isolated from 37 Mallards (21 from Lithuania, 8 from Russia, 6 from Belarus, and 2 from Latvia) were subjected to sequencing of the *ITS1* region. Based on DNA analysis, *S*. *rileyi* was confirmed in all tested birds. By comparing the infection rates of macrocysts in Mallards in Lithuania, significant differences were observed in different years (*p* = 0.036), and a significantly higher prevalence of infection was established in November–December than in September–October (*p* = 0.028). Given the amount of data per decade on the prevalence of *S*. *rileyi*, awareness of infection needs to be increased.

## 1. Introduction

The genus *Sarcocystis* is a large group of worldwide distributed protozoan parasites that infect mammals, birds, and reptiles. These parasites have an obligatory two-host life cycle [1]. The intermediate host gets infected through food or water-containing sporocysts, whereas the definitive host becomes infected through predating or scavenging tissues containing mature sarcocysts [1,2]. 

Wild and domestic birds are intermediate hosts for 28 *Sarcocystis* species [3,4]. Some of these species are pathogenic, causing severe myositis, encephalitis, hepatitis, and pneumonia [5,6,7,8,9,10]. Birds of the order Anseriformes are known to be intermediate hosts of five *Sarcocystis* species: *Sarcocystis albifronsi*, *Sarcocystis anasi*, *Sarcocystis platyrhynchosi*, *Sarcocystis rileyi*, and *Sarcocystis wobeseri* [4,11,12,13,14]. Four of these species, *S*. *albifronsi*, *S*. *anasi*, *S*. *rileyi*, and *S*. *wobeseri*, have been detected in ducks and geese from Lithuania [12,13,14,15]. Only macrocysts of *S*. *rileyi* are visible to the naked eye [11,14,15,16], while other *Sarcocystis* species form microcysts in the muscles of anseriforms [4,12,13]. Although *S*. *rileyi* is not considered to be pathogenic, hunted birds infected with macrocysts of this species are not suitable for human consumption [17,18,19,20,21,22]. Therefore, extensive efforts have been made to investigate the epidemiology and determine hosts of *S*. *rileyi*. 

For a long time, reports of macrocysts in the muscles of anseriforms came almost exclusively from North America. Macrocysts were observed in numerous duck species of the genera *Aix*, *Anas*, *Aythya*, *Bucephala*, *Mareca*, *Melanitta*, and *Spatula* [11,16,18,23,24,25,26,27,28,29,30,31,32,33,34]. Based on the first summary list of *Sarcocystis* spp. in birds, presented by Erickson in 1940, macrocysts of *Sarcocystis* spp. were detected in the Mallard (*Anas platyrhynchos*), American Black Duck (*Anas rubripes*), Gadwall (*Mareca strepera*), Northern Pintail (*Anas acuta*), Blue-Winged Teal (*Spatula discors*), and Northern Shoveler (*Spatula clypeata*) in North America [24]. Later in North America, macrocysts were recorded in the Green-Winged Teal (*Anas carolinensis*), Wood Duck (*Aix sponsa*), Greater Scaup (*Aythya marila*), Bufflehead (*Bucephala albeola*), American Wigeon (*Mareca americana*), Redhead (*Aythya americana*), Canvasback (*Aythya valisineria*), Common Goldeneye (*Bucephala clangula*) [26], Mottled Duck (*Anas fulvigula*), Lesser Scaup (*Aythya affinis*) [27], and Velvet Scoter (*Melanitta fusca*) [30]. 

In 2003, a detailed morphological characterization of macrocysts found in the Shoveler Duck (*Anus cylpeata*) was provided using light microscopy and transmission electron microscopy, and the parasite detected was redescribed as *S*. *rileyi* [11]. The first molecular characterization of *S*. *rileyi* was carried out in 2010 on macrocysts isolated from the Mallard collected in the USA [16]. Based on DNA sequencing of three genetic loci, macrocysts found in Mallards were confirmed as *S*. *rileyi* in Europe in 2011 [14]. Notably, reports on macrocysts in waterfowl, mainly Mallards, have increased in the twenty-first century in Europe [14,15,17,19,20,21,22,35,36]. 

Based on the experimental infection, the striped skunk (*Mephitis mephitis*) (Mephistidae family) was confirmed to be a definitive host of *S*. *rileyi* in North America [37,38]. The striped skunk is unsuitable for the distribution of *S*. *rileyi* in Europe since this small predatory animal lives on this continent only in captivity [39]. The molecular analysis enabled the identification of *S*. *rileyi* sporocysts in representatives of the families Canidae and Mustelidae in Europe. In Germany, *S*. *rileyi* was confirmed in the red fox (*Vulpes vulpes*) and in the raccoon dog (*Nyctereutes procyonoides*) [40]. In addition to these two predatory species, *S*. *rileyi* was also identified in the small intestines of the members of the family Mustelidae, in the American mink (*Neogale vison*), the European polecat (*Mustela putorius*), and the European badger (*Meles meles*) from Lithuania [41,42]. 

The prevalence of macrocysts of *Sarcocystis* in wild ducks depends on various factors, such as bird species, age, sex, location, etc., and has been broadly studied in North America [27,28,30]. However, detailed studies on *Sarcocystis* species identification, variety of intermediate hosts, life cycle, and infection rates of macrocysts in anseriforms in Europe are still lacking. Results of the first long-term (over a decade) surveillance study on macrocysts of *Sarcocystis* in wildfowl species in Europe (with more than 3000 anseriforms checked for macrocysts in Lithuania, Latvia, Russia, and Belarus) and molecular identification of *S*. *rileyi* in Mallards from four countries are presented in the present paper. 

## 2. Materials and Methods

### 2.1. Sample Collection

For the evaluation of macrocysts in wildfowl, 2649 birds were collected in Lithuania in 2011–2014 and 2017–2022. Mallards accounted for the largest number of birds surveyed (2362), although 287 wildfowl belonging to other 12 species were also analyzed. The examined birds were collected from all counties in Lithuania. 

The majority of the analyzed birds were legally hunted during the open hunting season (August–December) by licensed third parties, following the Hunting Law of the Republic of Lithuania. It should be noted that the birds were not hunted for the survey. Specifically, all examined Mallards, as well as 88 Eurasian Teals (*Anas crecca*), 46 Greater White-Fronted Geese (*Anser albifrons*), 29 Tufted Ducks (*Aythya fuligula*), 25 Garganeys (*Spatula querquedula*), 16 Common Goldeneyes, five Barnacle Geese (*Branta leucopsis*), and a single Bean Goose (*Anser fabalis*) were hunted. Furthermore, birds of several other wildfowl species found dead were received from Kaunas Tadas Ivanauskas Zoology Museum (the national authority responsible for monitoring wild birds found dead) and examined. This sample included 42 Velvet Scoters, 14 Gadwalls, 10 Long-Tailed Ducks (*Clangula hyemalis*), 10 Eurasian Wigeons (*Mareca penelope*), and a single Common Pochard (*Aythya ferina*). Carcasses of birds collected by hunters and staff from the Kaunas Tadas Ivanauskas Zoology Museum were frozen and delivered to scientific laboratories for examination of sarcocysts.

In addition, a total of 619 Mallards were received from neighboring countries. Specifically, 319 Mallards were delivered from the Kaliningrad Region of Russia, 202 birds came from Belarus, and 98 were sent from Latvia. All analyzed Mallards were hunted in the autumn seasons of 2012–2014 in accordance with the national hunting regulations valid in the countries concerned and checked for macrocysts of *Sarcocystis*. 

The skin of all collected birds was peeled from the head to the area of the knee tendons, and the muscles were examined grossly for macrocysts of *Sarcocystis* spp. visible to the naked eye. 

### 2.2. Molecular Examination of Macrocysts

During the study, the investigators presumed that macrocysts observed in ducks belonged to *S*. *rileyi*. For the confirmation of the parasite species, only some of the macrocysts isolated from the infected birds were subjected to DNA analysis. *Sarcocystis* species have not been molecularly identified in all infected ducks, mainly due to the fact that the research was carried out by two independent research groups and also taking financial issues into account. 

During the period of 2011–2014, birds examined were transported to the Laboratory of Molecular Ecology of the Nature Research Centre (Vilnius, Lithuania). At that time, macrocysts isolated from 32 infected Mallards were subjected to DNA analysis for confirmation of *Sarcocystis* species. In all cases, a single macrocyst was excised from an individual bird. In total, two of the infected Mallards came from Latvia, six from Belarus, eight from the Kaliningrad Region of Russia, and the remaining sixteen birds were hunted in Lithuania. 

Meanwhile, from 2017 to 2022, monitoring of macroscopic sarcocysts found in ducks hunted in Lithuania was carried out. The collected individuals of wildfowl species were submitted to the Laboratory of Forest Sciences Department of the Faculty of Forestry and Ecology of the Agriculture Academy of Vytautas Magnus University (Kaunas, Lithuania). The carcasses of birds were surveyed for the macrocysts of *Sarcocystis*, but only five macrocysts isolated from individual Mallards hunted in late autumn 2022 in Lithuania were examined by molecular methods. Thus, in total, macrocysts isolated from 37 Mallards were used for molecular identification of *Sarcocystis* species. 

Prior to the molecular analysis, the macrocysts were stored in individual tubes filled with 75% ethyl alcohol at −20 °C. The genomic DNA from the sarcocysts was extracted using spin-column based commercial kits, “NucleoSpin Tissue, Mini kit” (Macherey-Nagel, Düren, Germany), “QIAamp^®^ DNA Micro Kit” (Qiagen, Hilden, Germany), or “GeneJET Genomic DNA Purification Kit” (Thermo Fisher Scientific Baltics, Vilnius, Lithuania). Regardless of the kit, 100 μL of commercial Elution Buffer was added in the last step of DNA purification to elute the genomic DNA. For *Sarcocystis* species identification, all the DNA samples were subjected to the amplification of internal transcribed spacer 1 (*ITS1*), situated between *18S* rRNA and *5*.*8S* rRNA. This molecular locus is considered to be the most variable for *Sarcocystis* spp. in birds [43]. Furthermore, eight DNA isolates obtained from macrocysts of Mallards hunted in Lithuania were genetically characterized at *18S* rDNA and *28S* rDNA, and four of these isolates were also characterized at mitochondrial cytochrome c oxidase I (*cox1*) and the RNA polymerase B gene of the apicoplast genome (*rpoB*). The oligonucleotide primers used for the amplification, conditions of PCRs, purification of amplified PCR products, and procedures of direct Sanger sequencing were performed as described previously [44]. The resulting sequences were manually checked for ambiguously placed nucleotides and merged into a single fragment in the case of *18S* rDNA. Finally, the obtained DNA fragments were checked for similarity using the nucleotide BLAST program [45]. 

### 2.3. Statistical Data Analysis

A 95% confidence interval (CI) for the determined prevalence of macrocysts in different years, host species, countries, sexes, and months was computed using Sterne’s exact method [46]. A pair-wise comparison of infection rates of macrocysts was made by the unconditional exact test [47]. The following analysis is more sensitive in detecting differences than Fisher’s exact test and is especially advantageous in the case of small samples (*n1*, *n2* < 100). Fisher’s exact test was used to compare the prevalence data of macrocysts between three and six samples, and the Chi-squared test was used when more than six samples were compared. Statistical analyses were performed in Quantitative Parasitology 3.0 software [48]. 

## 3. Results

### 3.1. The Occurrence of Macrocysts in Wildfowl Species and Parasite Load

Macroscopic sarcocysts were found almost exclusively in the pectoral muscles of birds, although they were also detected in the neck muscles of several individuals. The exact number of birds with macrocysts in their necks was not recorded.

**Figure 1:**
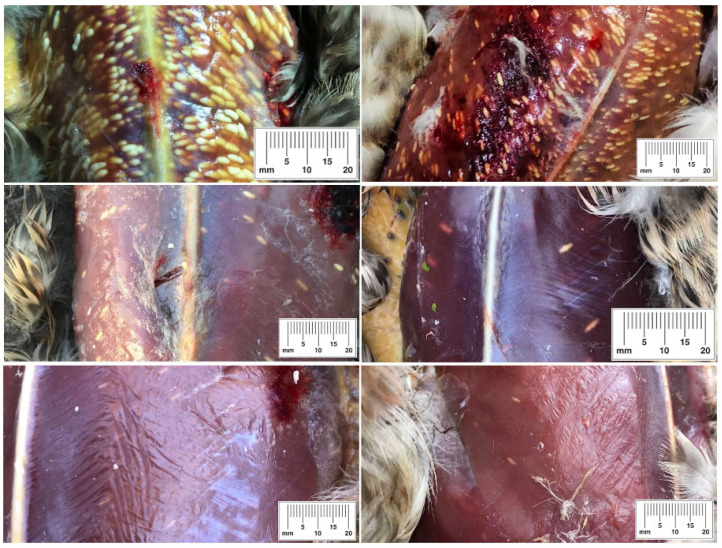
Different parasite loads of macrocysts of *Sarcocystis* in Mallard carcasses. Images are ordered by the size of the parasite load, from highest to lowest. The scale shows the size of macrocysts in mm.

Macrocysts of *Sarcocystis* were yellowish-white in color and 2.0–7.0 × 1.5–2.0 mm (1.8 × 4.6 mm) in size. They resembled rice grains in shape and color. It is noteworthy that the parasite load varied greatly in infected birds. Among all the examined individuals, several were heavily infected, some birds acquired moderate infection, and in the carcasses of other birds, only single macrocysts were detected (Figure 1). However, during this study, the parasite load in the muscles of birds was not examined in detail, and parasite load categorization according to the number of macrocysts observed was subjective. 

### 3.2. The Prevalence of Macrocysts of Sarcocystis in Wildfowl in Lithuania and Neighboring Countries

Overall, macrocysts were found in 206 of 2362 (8.7%, CI = 7.65–9.93%) Mallards collected in Lithuania during different years (2011–2014 and 2017–2022). A similar prevalence was observed in the examined Mallards collected in Lithuania, the Kaliningrad Region of the Russian Federation, Belarus, and Latvia, from 3.1% (CI = 0.84–8.53%) in Latvia to 5.9% (CI = 3.37–10.08%) in Belarus (Table 1), although differences were not significant. However, significant differences were noticed when comparing the prevalence of infection in 2012 (*p* = 0.005) and 2014 (*p* = 0.002). In 2012, infection rates were much higher in the Kaliningrad Region of Russia (*p* = 0.002) and Belarus (*p* = 0.010) than those in Lithuania. Whereas in 2014, a significantly higher prevalence of macrocysts was recorded in Lithuania than that observed in the Kaliningrad Region of Russia (*p* = 0.005) and Belarus (*p* = 0.008) (Table 1). 

**Figure 2:**
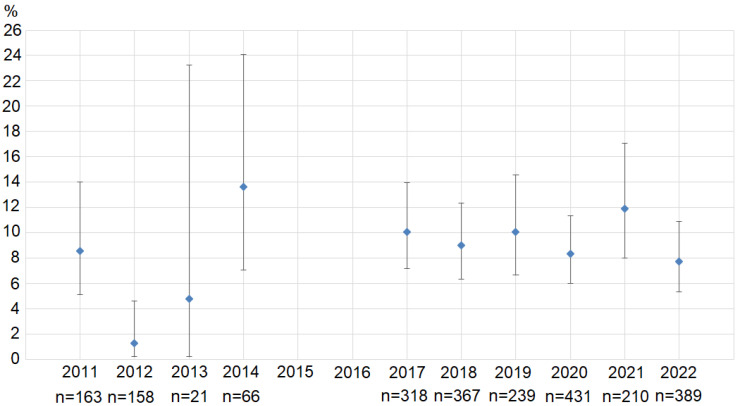
Prevalence of macrocysts in Mallards collected in Lithuania in August–December 2011–2014 and 2017–2022. Error bars indicate 95% confidence intervals. *n* shows the size of the sample in each analyzed year.

A significant difference (χ^2^ = 17.97, df = 9, *p* = 0.035) was observed when comparing the prevalence of macrocysts in Mallards collected in different years (2011–2014 and 2017–2022) in Lithuania (Figure 2). The difference remained significant (χ^2^ = 15.70, df = 7, *p* = 0.028) when samples containing fewer than 150 birds (2013 and 2014 years) were excluded. The lowest prevalence was estimated in 2012 (1.3%, CI = 0.23–4.60%), while the highest was in 2014 (13.6%, CI = 7.07–24.01%) (Figure 2). Prevalence was relatively consistent, varying from 7.7 to 11.9% in 2017–2022. The prevalence of macrocysts in 2012 was significantly lower than that determined in 2011 (*p* = 0.002), 2014 (*p* < 0.001), 2017 (*p* < 0.001), 2018 (*p* < 0.001), 2019 (*p* < 0.001), 2020 (*p* = 0.001), 2021 (*p* < 0.001), and 2022 (*p* = 0.002). Also, a low prevalence was determined in Mallards collected in 2013 (4.8%, CI = 0.2–23.3%); however, this data should be interpreted with caution due to the small sample size of the birds examined (*n =* 21). 

Out of the other 12 wildfowl species tested (287 individuals), macrocysts were detected only in the breast muscles of the Eurasian Teal, with a low prevalence (2.3%, 2 infected of 88 analyzed, CI = 0.41–7.79%). In Lithuania, a significantly higher (*p* = 0.030) prevalence of macrocysts was established in Mallards (206/2362, 8.7%) than that observed in Eurasian Teals (2/88, 2.3%).

### 3.3. Factors Influencing the Prevalence of Macrocysts of Sarcocystis in the Mallard

**Figure 3:**
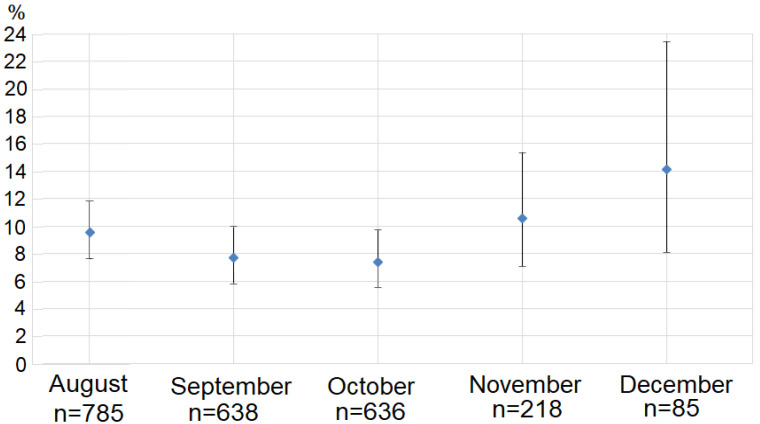
Monthly prevalence of macrocysts infection in Mallards in Lithuania from August to December. Dots represent the percentage infection rates. Error bars indicate 95% confidence intervals of prevalence. *n* indicates the sample size in the months studied.

In 2011–2014 and 2017–2022, the lowest prevalence of macrocysts of *Sarcocystis* spp. in Mallards in Lithuania was noticed in October (7.4%, CI = 5.56–9.72%) and in September (7.7%, CI = 5.78–10.01%) (Figure 3), while the highest was observed in December (14.1%, CI = 8.07–23.40%). The infection rate in August (9.6%, CI = 7.63–11.83%) and in November (10.6%, CI = 7.05–15.31%) was similar. The prevalence was independent of the month when Mallards were hunted. However, a pair-wise analysis demonstrated a significantly higher prevalence in November–December than in September–October (*p* = 0.028). Also, a significantly higher prevalence of macrocysts was determined in November–December than in October (*p* = 0.036) and in December than in September–October (*p* = 0.038).

In Lithuania, during the hunting season (2011–2014 and 2017–2022), a higher but statistically insignificant prevalence was detected in Mallard males (99 infected of 1085 analyzed, 9.1%, CI = 7.55–10.99) than in females (107 infected of 1277 examined, 8.4%, CI = 6.98–10.03).

### 3.4. Molecular Identification of S. rileyi in Mallards from Lithuania, Russia, Belarus, and Latvia

Based on the comparison of the current work-generated DNA sequences, *S*. *rileyi* was confirmed in all tested Mallards, 21 from Lithuania, eight from the Kaliningrad Region of Russia, six from Belarus, and two from Latvia. Comparing the present work determined sequences of the five genetic loci, intraspecific variation was not observed.

Thirty-seven 942 bp-long sequences of the complete *ITS1* region were 100% identical to each other and showed 100% identity to *S*. *rileyi* previously identified in Mallard from Lithuania (HM185744) and to other European isolates of *S*. *rileyi* from ducks (KJ396584, MZ151434, MZ468639-40, LT992314). Single *ITS1* region sequences from four different countries have been submitted to GenBank under accession numbers OR416415–OR416418. The obtained *ITS1* sequences also displayed 99.9% identity to *S*. *rileyi* from Mallard collected in the USA (GU188427) and 99.5–100% identity to *S*. *rileyi* observed in the small intestines of the American mink, European polecat, and European badger (OP970971–81) from Lithuania. 

In addition to the *ITS1* region, eight partial 1753 bp-long *18S* rDNA, eight 1499 bp-long *28S* rDNA, four 1053 bp-long *cox1*, and four 762 bp-long *rpoB* sequences were identified. As the sequences identified during this study were identical in the same genetic region, one each of *18S* rDNA (OR416136), *28S* rDNA (OR416169), *cox1* (OR423018), and *rpoB* (OR423019) were uploaded to the GenBank. In the current work, the obtained rDNA sequences were 100% identical with homological sequences of *S*. *rileyi* previously defined in Mallard from Lithuania (HM185742–43). Our 1753 bp-long *18S* rDNA sequences showed 100% identity with *S*. *rileyi* isolated from various countries and hosts (GU120092, KJ396583, KM233682, MZ151434, MZ468637–38), 99.8% identity with *S*. *rileyi* isolates obtained from Mallard, Gadwall European and Eurasian Wigeons in the UK (LT992317–23), and 99.8% identity with the 1010 bp-long sequence of *S*. *rileyi* (KX170885) obtained from Northern Shoveler in Mexico. Notably, sequence mismatches were observed only at the ends of the aligned *18S* rDNA sequences, suggesting that these differences are due to sequencing errors rather than representing intraspecific genetic variability. The 1499 bp-long *28S* rDNA sequences determined in the present work demonstrated 100% identity to *S*. *rileyi* from Mallards in Denmark (MZ151434, MZ468640–41) from Mallard in the USA (GU188426) and 99.9% identity to *S*. *rileyi* from Common Eider in Norway (KJ396585). The 762 bp-long *rpoB* sequence determined in the present study showed 100% identity with that of *S*. *rileyi*, previously also isolated from Mallard in Lithuania (MF596308). Finally, our 1053 bp-long *cox1* sequence displayed 100% similarity compared to *S*. *rileyi* from Common Eider in Norway (KJ396582). 

In terms of interspecific genetic variability, the largest differences between *S*. *rileyi* and the most related species, *Sarcocystis atraii*, *Sarcocystis wenzeli*, *Sarcocystis cristata*, *Sarcocystis chloropusae*, *S*. *anasi* and *S*. *albifronsi* [3,49,50], were observed in the *ITS1* region (9.9–25.7%). Comparing *S*. *rileyi* with the listed *Sarcocystis* species, much less variability was determined within *cox1* (2.4–3.6%), *rpoB* (2.6–4.1%) and *28S* rDNA (0.6–3.8%) while differences in *18S* rDNA were minimal (0.3–1.1%). 

## 4. Discussion

### 4.1. The Genetic Identification and Variability of S. rileyi

Among hunters and veterinarians, *S*. *rileyi* is known as a parasite that forms “rice-like grains”. Macrocysts were discovered in a duck by Riley and were named *S*. *rileyi* by Stiles in 1893 [51]. Thus, *S*. *rileyi* is the first *Sarcocystis* species discovered in birds and, in general, one of the first *Sarcocystis* species described [1]. Researchers finding macrocysts in the muscles of waterfowl attributed them to *S*. *rileyi* [11,23,24,26]. Taking into account that five *Sarcocystis* spp. are known to form sarcocysts in the muscles of anseriforms [4], the possibility that more than one species of *Sarcocystis* may constitute macrocysts in the muscles of ducks cannot be excluded [16]. Therefore, molecular methods are needed for the identification of *S*. *rileyi* [11,16]. In the current investigation, macrocysts were found in 239 individuals of two wildfowl species (237 Mallards and two Eurasian Teals). Due to the limitation of this study that molecular methods were only used on the part of the infected birds, we cannot claim that all infected ducks harbored macrocysts of *S*. *rileyi*. Overall, thirty-seven macrocysts were molecularly analyzed, and *S*. *rileyi* was identified in twenty-one Mallards from Lithuania, eight Mallards from Russia’s Kaliningrad Region, six Mallards from Belarus, and two Mallards from Latvia. To the best of our knowledge, this is the first report of *S*. *rileyi* in Russia, Belarus, and Latvia. Notably, macrocysts have previously been found in the muscles of ducks from Russia, but no molecular investigations have been carried out on the parasites detected [52,53]. Based on the recent reports on *S*. *rileyi* in various European countries [14,15,17,19,20,21,22,35,36] and records of experienced local hunters, macrocysts of *Sarcocystis* are assumed to be spreading geographically [22]. In Lithuania, *S*. *rileyi* in Mallards has been previously confirmed using DNA sequence analysis [14,15]. Summarizing the results of this research and previous studies carried out in Lithuania, all 40 genetically examined macrocysts isolated from different Mallard individuals were identified as *S*. *rileyi* [14,15; and present work]. In light of these data, we believe that most of the macrocysts found in the muscles of Mallards hunted in Lithuania should belong to *S*. *rileyi*. 

Based on the results of the present and earlier studies [14,15,16,21,22,43,54], the *ITS1* region is the most suitable loci for the identification of *S*. *rileyi*, as significantly less interspecific variation is observed when comparing nuclear sequences of the *18S* rDNA, *28S* rDNA, mitochondrial *cox1*, and apicoplast *rpoB* loci. Recently, the complete *ITS2* region of *S*. *rileyi*, which is located between *5*.*8S* rDNA and *28S* rDNA, has been reported [22]. This genetic marker has good potential for the identification of species in the Sarcocystidae family [55]. In the current study, no intraspecific variation has been observed when analyzing sequenced isolates of *S*. *rileyi*. The restricted genetic diversity of *S*. *rileyi* has been reported previously [15]. Among the genetic regions currently used most frequently for the characterization of *Sarcocystis* species employing birds as intermediate hosts [3,56], *ITS1* is known to be the most variable at the intraspecific level [57]. The current data available in GenBank show low intraspecific variability of *S*. *rileyi* within *ITS1*. Intriguingly, high intraspecific genetic variation was observed for *Sarcocystis halieti* [54,58] and *Sarcocystis falcatula* [59,60,61] using birds of several different orders as their intermediate hosts. Thus, the relatively low intraspecific genetic variability of *S*. *rileyi* might be explained by the high intermediate host specificity, as macrocysts of *S*. *rileyi* have only been validated in ducks of the family Anatidae [11,14,15,16,17,18,19,20,21,22].

### 4.2. The Distribution of Sarcocystis Macrocysts in Different Wildfowl Species

Summarizing record data on macrocysts of *Sarcocystis* spp. in wildfowl species in North America, this infection occurs significantly more often in dabbling ducks than in diving ones [27,32]. Dabbling ducks feed in shallower wetlands and upland regions, so they have a greater chance of ingesting the fecal material of predators than divers do [28]. Apart from North America, macrocysts have been found in ducks in Europe [14,15,17,19,20,21,22,35,36,62] but not in Australia or Asia [63,64], while ducks were not examined for *Sarcocystis* spp. in detail in South America and Africa [1,11,16]. 

The range of intermediate hosts of macrocysts of *Sarcocystis* is considerably smaller in Europe than in North America. In Europe, macrocysts were mostly recorded in Mallards [14,17,19,20,21,22,35,62], Common Eider (*Somateria mollissima*) in Norway [36], Eurasian Wigeon, Eurasian Teal in Finland and the UK [15,21], in the Gadwall, and Northern Pintail in the UK [21]. In the present study, macrocysts were observed in Mallards and Eurasian Teals collected in Lithuania. This is the first survey in which macrocysts of *Sarcocystis* have been detected in European Teal Lithuania. In the previous study conducted in Lithuania, macrocysts were found only in Mallards [14,15,62]. In the present study, macrocysts were not noticed in other wildfowl species analyzed. The non-detection of macrocysts in other bird species can be explained by the small number of individuals examined (*n* = 1–46). However, taking into account records of macrocysts and data on infection rates in this study and previous investigations [14,15,21], it is suggested that the Mallard is the main source of macrocyst infection in Europe. Nevertheless, it cannot be ruled out that the highest number of detections of macrocysts in Mallards in Europe [15,21] is related to the fact that this species is the most hunted of all the anseriforms [65,66]. On the contrary, in North America, the prevalence of macrocysts in Mallards was not the highest as compared to that in Northern Pintails, Blue-winged Teals, Green-Winged Teals, American Wigeons, Northern Shovelers [27,28], Lesser Scaups, Velvet Scoters [30], American Black Ducks [32], and Mottled Ducks [34]. Thus, in North America, compared to Europe, macrocysts are more common in various duck species [11,16,18,23,24,25,26,27,28,29,30,31,32,33,34]. A smaller range of intermediate hosts in Europe, compared to North America, may indicate that in Europe, the infection of macrocysts of *Sarcocystis* spp. has spread recently [14,15,17,19,20,21,22,35,36,62].

### 4.3. Factors Affecting the Prevalence of Macrocysts of Sarcocystis spp. in the Mallard in Lithuania

The first report on macrocysts in Mallards in Europe was recorded in Russia in the middle of the 20th century [52,53]. Reports of macrocysts of *Sarcocystis* in Europe have increased in the 21st century. Macrocysts in ducks were detected in Lithuania [14,15,62], Poland [17], the Slovak Republic [35], Finland [15], Norway [36], the Czech Republic, Sweden, the Netherlands, Austria [for details see 15], Hungary [20], UK [19,21], and Denmark [22]. Thus, macrocysts were most commonly reported in northern, eastern, and central Europe. Based on molecular results, the invasive American mink is one of the definitive hosts of *S*. *rileyi* [42]. This predator of the family Mustelidae is native to North America, and its introduced range [67,68] partially coincides with the geography of the distribution of macrocysts of *Sarcocystis* spp. in Europe. Therefore, it can be speculated that the spread of *S*. *rileyi* in Europe is associated with the introduction of American mink to this continent.

In the present study, the exceptionally low prevalence of macrocysts (1.3%, CI = 0.23–4.60%) was determined in Mallards hunted in 2012 in Lithuania (Figure 3). Infection rates determined in 2012 were significantly lower than those observed in 2011, 2014, and 2017–2022. Furthermore, infection rates of macrocysts of *Sarcocystis* spp. observed in Lithuania and Kaliningrad/Belarus differed significantly in 2012 and 2014 (Table 1). These findings can be partly accounted for by the different periods when Mallards were hunted in the countries concerned. In Lithuania, the majority of the ducks analyzed in 2012 were hunted in August–early September (with local breeders forming the majority of the analyzed ducks), whereas in Belarus and the Kaliningrad Region of Russia, ducks were hunted in August–December 2012. Some Mallards staging and wintering in Lithuania, Belarus, and the Kaliningrad Region of Russia in October–December are breeders from Northern Europe [69]. A higher prevalence of macrocysts detected in Belarus and the Kaliningrad Region of Russia in 2012 is likely to have been caused by a larger proportion of the analyzed migrants from the major region of Northern Europe [70,71]. It should be noted that a different prevalence of macrocysts was also observed in different geographical areas of the USA and Canada [27,30]. The data obtained was explained by the different migratory routes of the infected birds and the abundance of predators in the breeding and wintering grounds of ducks [30]. 

To the best of our knowledge, the current study is the first to evaluate the prevalence of macrocysts of *Sarcocystis* spp. in intermediate hosts in different months. We demonstrated that the infection rate of macrocysts in Mallards hunted in Lithuania was significantly higher in November–December than in Mallards hunted in September–October. The data obtained can be explained by the fact that in November–December, migrants from Northern Europe form a significant proportion of all Mallards recorded in Lithuania [65,66]. However, to test the above assumption, further research on this infection is needed by comparing epidemiological data on parasites with genetic data on Mallard populations. 

In this study, we found no significant differences when comparing the detection of macrocysts in the muscles of female and male Mallards. Thus, our findings are in agreement with previous studies showing that the prevalence of macrocysts does not depend on the sex of the birds examined [27,30]. 

### 4.4. The Significance of Macrocyst Infection in Ducks

Previous studies have demonstrated that some ducks infected with macrocysts of *Sarcocystis* show signs of myopathy [5,6,7,8,9,10]; however, histological examination does not show that the muscles of ducks are severely affected [21]. Even though this infection is not considered pathogenic, it may interfere with the energy and protein metabolism of birds [33]. Furthermore, severe infections of macrocysts in muscles used for locomotion may cause a reduced flying capacity and ability to withstand migration, weakness, and an increased probability of becoming a victim of predators [72]. 

Significant economic losses are incurred worldwide as a result of macrocysts of *Sarcocystis* spp. [21]. The Mallard is the world’s most abundant and widely distributed dabbling duck species, whose global population is estimated at 20 million individuals [66]. In Lithuania, it is the most abundant breeding wildfowl species, with an estimated population of about 60,000 pairs [71]. The Mallard is one of the most harvested game species worldwide, with more than 4.5 million birds annually hunted in Europe [65]. Activities related to the hunting of the Mallard constitute an important part of income in the recreation, tourism, agriculture, food, and catering sectors. Meat from hunted ducks infected with macrocysts of *Sarcocystis* is not suitable for human consumption [1,15,19,21,22,35,36]. The toxic effects of the parasites can be harmful when eating heavily infected ducks [30], though properly cooked meat from infected ducks is considered to be harmless to humans [73]. Therefore, awareness related to the infection of macrocysts must be increased among hunters, veterinarians, and the general public. Infection by *Sarcocystis* spp. parasites and their prevention are mentioned among the biological contamination risk factors specified in the game meat safety requirements valid in Lithuania and in other countries of the European Union. There is a lack of such strict regulations in the countries of Eastern Europe, where large quantities of meat from hunted ducks are consumed in the households of hunters. Furthermore, changes in game eating habits are observed; dishes are increasingly being prepared from raw, uncooked game meat. Therefore, hunters should be able to recognize macroscopic sarcocysts and know how to utilize the infected birds [19,21,22].

The Mallard can also form a potential link between domestic birds and wild ducks in spreading parasites of the genus *Sarcocystis*. During migration periods, staging Mallards are recorded in numerous pools and ponds used for the production of domestic ducks and geese in various regions of Eastern Europe [71]. These ducks are very tolerant of human presence and are common in urban and agricultural habitats, thus forming a potential link between wild waterfowl, poultry, and humans [74]. More than three million Mallards reared in captivity are released annually for hunting purposes in Europe [75]. There is a lack of knowledge about the infection of macrocysts of *Sarcocystis* in poultry, as reports on macrocysts in domestic ducks are very scarce [24,53]. Therefore, it is important to undertake studies into the potential infection of macrocysts in poultry, particularly among the millions of farmed Mallards that are released for hunting purposes in Europe each year.

## 5. Conclusions

*Sarcocystis rileyi* was, for the first time, identified in Mallards hunted in the Kaliningrad region of the Russian Federation, Belarus, and Latvia based on DNA sequencing analysis of some of the birds infected with macrocysts. Findings of the present work show that infection rates of macrocysts of *Sarcocystis* in the muscles of ducks depend on the host species, hunting year, geographical region, and hunting season. 

The Mallard is the main source of infection by macrocysts in Europe. Since the Mallard is one of the most harvested game bird species worldwide and its meat contaminated with macrocysts of *Sarcocystis* is not suitable for consumption, awareness of the infection of macrocysts must be increased. Hunted ducks should be tested for rice-like macrocysts, and it is recommended that hunted macrocyst-infected ducks not be left in the wild, consumed as food, or fed to domestic predators. Also, further studies on the macrocysts of *Sarcocystis* spp. in farmed ducks are needed.

## Figures and Tables

**Table 1:** The prevalence of macrocysts in examined Mallards collected in four countries in 2012–2014.

Year	Lithuania	Kaliningrad Region (Russia)	Belarus	Latvia
2012	2/158 (1.3%) ^b,e^	10/107 (9.4%) ^c,f^	6/72 (8.3%) ^g^	3/98 (3.1%)
2013	1/21 (4.8%)	2/74 (2.7%)	5/62 (8.1%)	
2014	9/66 (13.6%) ^a,h^	4/138 (2.9%) ^d,i^	1/68 (1.5%) ^j^	
Overall	12/245 (4.9%)	16/319 (5.0%)	12/202 (5.9%)	3/98 (3.1%)

The statistically significant *p* values (*p* < 0.05) obtained by comparing the prevalence of macrocysts in four countries in different years are given below. ^a^ > ^b^ *p* < 0.001; ^c^ > ^d^ *p* = 0.034; ^f^ > ^e^ *p* = 0.002; ^g^ > ^e^ *p* = 0.010; ^h^ > ^i^ *p* = 0.005; ^h^ > ^j^ *p* = 0.008.

## Data Availability

The sequences of *S*. *rileyi* generated in the current research were submitted to the NCBI GenBank database under accession numbers OR416136, OR416169, OR416415–OR416418, OR423018, OR423019.
